# RNA sequencing describes both population structure and plasticity-selection dynamics in a non-model fish

**DOI:** 10.1186/s12864-021-07592-4

**Published:** 2021-04-15

**Authors:** Matt J. Thorstensen, Melinda R. Baerwald, Ken M. Jeffries

**Affiliations:** 1 Department of Biological Sciences, University of Manitoba, Winnipeg, MB R3T 2N2 Canada; 2 California Department of Water Resources, West Sacramento, CA 95691 USA

**Keywords:** Transcriptomics, Microsatellites, Evolution, Plasticity, Population genetics, Outlier test, Selection, Adaptation

## Abstract

**Background:**

Messenger RNA sequencing is becoming more common in studies of non-model species and is most often used for gene expression-based investigations. However, the method holds potential for numerous other applications as well—including analyses of alternative splicing, population structure, and signatures of selection. To maximize the utility of mRNA data sets, distinct analyses may be combined such as by exploring dynamics between gene expression with signatures of selection in the context of population structure. Here, we compare two published data sets describing two populations of a minnow species endemic to the San Francisco Estuary (Sacramento splittail, *Pogonichthys macrolepidotus*): a microsatellite data set showing population structure, and an mRNA whole transcriptome data set obtained after the two populations were exposed to a salinity challenge. We compared measures of population structure and genetic variation using single nucleotide polymorphisms (SNPs) called from mRNA from the whole transcriptome sequencing study with those patterns determined from microsatellites. For investigating plasticity and evolution, intra- and inter-population transcriptome plasticity was investigated with differential gene expression, differential exon usage, and gene expression variation. Outlier SNP analysis was also performed on the mRNA data set and signatures of selection and phenotypic plasticity were investigated on an individual-gene basis.

**Results:**

We found that mRNA sequencing revealed patterns of population structure consistent with those found with microsatellites, but with lower magnitudes of genetic variation and population differentiation consistent with widespread purifying selection expected when using mRNA. In addition, within individual genes, phenotypic plasticity or signatures of selection were found in almost mutual exclusion (except *heatr6*, *nfu1*, *slc22a6*, *sya*, and *mmp13*).

**Conclusions:**

These results show that an mRNA sequencing data set may have multiple uses, including describing population structure and for investigating the mechanistic interplay of evolution and plasticity in adaptation. MRNA sequencing thus complements traditional sequencing methods used for population genetics, in addition to its utility for describing phenotypic plasticity.

**Supplementary Information:**

The online version contains supplementary material available at 10.1186/s12864-021-07592-4.

## Background

As the cost of sequencing continues to come down, messenger RNA (mRNA) sequencing is becoming more affordable for studying non-model species, while providing transcriptome sequencing data on the order of tens of millions of reads per individual. The abundance of information in mRNA data allows investigators to pursue a variety of gene expression and genetic variation-based approaches. Using mRNA data, researchers may combine plasticity- and selection-focused approaches in the context of population structure; approaches which have implications for physiology, adaptive evolution, and conservation.

In wild, non-model species, descriptions of population structure can guide management decisions and work in tandem with studies on local adaptation [1, 2]. As an expressed molecule, messenger RNA may carry important information about functional genomic variation through *cis*-acting regulatory mechanisms under selection [3]. This selection may be informative for investigations on local adaptation and evolutionary patterns that help define evolutionarily significant units or conservation units, but can interfere with other objectives such as the delineation of management units or describing population subdivision [1, 4]. In particular, management units are defined by their demographic independence, and neutral markers are necessary for representing effective population sizes and demography [1]. Targeting synonymous single nucleotide polymorphisms (SNPs) may yield neutral markers using mRNA, because their non-functional nature may decrease the adaptive significance of these SNPs. However even synonymous SNPs may be widely under selection, such as from codon usage bias, and purifying selection is widespread throughout organisms’ transcriptomes [5–7]. Therefore, validation for neutral patterns may need to be performed with SNPs called from mRNA sequences before using them for studying population structure or when used for making conservation decisions.

Another challenge with using mRNA data for population structure is that of sample size. MRNA sequencing is expensive, partly because of the great sequencing depth required for transcript expression quantification, relative to DNA-based methods. In practice, sample sizes may be low for genetic data that are otherwise appropriate for physiological questions (e.g. *n* = 6–8 per experimental treatment) in mRNA sequencing studies. Two properties of mRNA used to study genetic variation may mitigate the issues of low sample sizes, however. First, SNPs called for genetic approaches may be drawn from combined treatment groups in physiological studies, if the overarching experimental design includes comparisons between populations [8, 9]. For example, in the present study, two populations of fish are compared, each with three experimental treatments. Because *n* = 14 individuals may be appropriate for estimating population allele frequencies, low sample sizes in mRNA studies may nevertheless yield informative population structure estimates [10]. The second factor that may mitigate sample size issues is that of the number of markers available in mRNA sequencing. Microsatellite-based studies often use 10–20 markers, SNP arrays contain several hundred to tens of thousands of markers, and reduced representation-based studies often have 10,000–200,000 markers. MRNA sequencing data can yield hundreds of thousands of SNP markers, similar in quantity to those produced by reduced representation approaches, and orders of magnitude above those provided by microsatellites or SNP arrays [9, 11–14]. This abundance of data allows for precise estimations of genetic variation and population structure, such as through bootstrapping of *F* statistics [4]. While several studies apply mRNA sequencing to population genetic approaches, concordant issues of widespread purifying selection in the transcriptome and sample size concerns suggest comparisons between mRNA- and established DNA-based methods are needed [8, 9, 12–15].

For studying phenotypic plasticity and genetic variation, a wide body of research on the topic explores plasticity in morphological or phenological traits [16, 17]. MRNA sequencing, however, provides an opportunity for researchers to study phenotypes defined by gene expression with respect to adaptive evolution [18–20]. In conjunction with signatures of selection across the transcriptome, mRNA sequencing has great potential for addressing the different roles of plasticity on evolution because of its dual uses in observing transcript quantification and genetic variation [20]. For example, divergence in plasticity likely contributes to adaptive responses to environmental change, while additivity and stability of *cis*-acting regulation has shown potential as a “substrate for the early stages of adaptive evolution” [3, 21]. A mechanistic view of plasticity expressed in individual genes may thus reveal the processes by which plasticity and evolution can enable populations to adapt to changing environments. The most well-characterized method for analyzing mRNA sequencing data for this plasticity is that of testing for differential gene expression (DGE) between groups of interest. Here, either laboratory studies investigate possible molecular mechanisms underlying some physiological parameter, such as those associated with climate change [22], or studies on wild-caught organisms provide evidence for environmental stressors that may affect a population’s viability [23]. Patterns of alternative splicing have been investigated using mRNA as well, revealing possible variation underlying adaptive radiations [24, 25], along with stress responses and acclimation associated with temperature [26, 27]. These data, represented by models describing differential exon usage (DEU), reveal patterns potentially hidden from DGE because exons may be differentially used under contrasting conditions, but the transcript overall may show little or no difference in abundance [28–30]. Recent advances in mRNA sequence alignment, such as by the SuperTranscript pipeline, have permitted the application of these methods to non-model species by using a de novo reference transcriptome against which to align data [30]. Gene expression variability (GEV) has also been described for analyzing mRNA sequencing data [31]. Here, variation from technical and biological origins are teased apart to investigate the role of expression variability in affecting physiological parameters, especially in the context of factors such as diet or age [31].

In the present study, we explored the potential for applying mRNA data to questions of population structure, phenotypic plasticity and evolution in the Sacramento splittail (*Pogonichthys macrolepidotus*) in the San Francisco Estuary, California, USA. There are two populations described in the species: the Central Valley population with an overall higher effective population size, and the San Pablo population which exists in a more saline environment and shows greater salinity tolerance and phenotypic plasticity when challenged with salinity [8, 32–34]. The role of mRNA sequencing for population genetic questions was investigated by comparing patterns of population structure and genetic variation between a published data set of microsatellites [33] and one using mRNA sequencing [8], with individuals sampled from the same locations at approximately the same times. Putatively neutral SNPs from mRNA were thus compared with microsatellites to assess the extent to which mRNA data may reflect population genetic patterns, in addition to a set of overall SNPs. Each data set contains individuals sampled from the same populations. In addition, within the mRNA data, the relationship between evolution and phenotypic plasticity in the form of DGE, DEU, and GEV is tested by observing signatures of selection and phenotypic plasticity in individual genes, as modeled by SuperTranscripts [30]. Here, we hypothesized that plasticity may diverge from adaptive variation within genes because plasticity plays a large role in the San Pablo population’s response to salinity; local adaptation may therefore have led to plastic gene expression rather than polymorphisms within transcripts and genes. Thus, we predicted that signatures of selection as identified by outlier SNPs would reside within genes not expressing any of DGE, DEU, or GEV.

## Results

### Population Structure & Genetic Variation

**Fig. 1:**
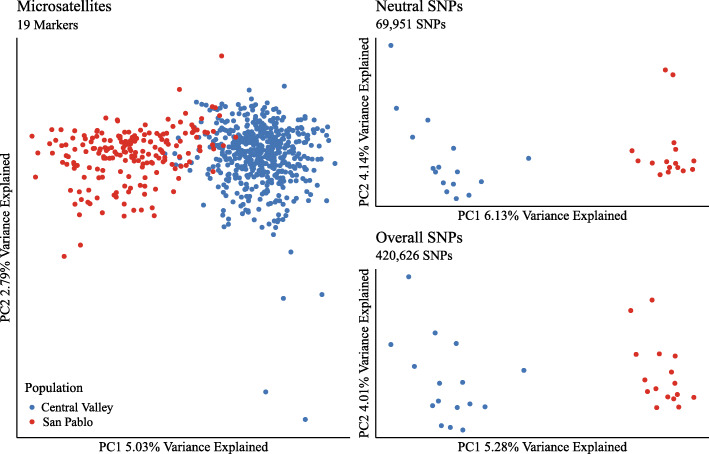
Principal component analysis of microsatellites, neutral SNPs from RNA, and overall SNPs from RNA for two populations of Sacramento splittail (*Pogonichthys macrolepidotus*). The Central Valley population represents an overall larger, less salinity-tolerant group than the San Pablo Bay population. Microsatellite data for the Central Valley population (*n* = 528) and the San Pablo population (*n* = 191) individuals, and are comprised of 19 markers with at least 80% present data, filtered for family structure where full-siblings were removed. Neutral and overall single nucleotide polymorphisms (SNPs) were generated with mRNA sequencing data of 32 fish (*n* = 16 per population). There were a total of 420,626 overall SNPs and 69,951 neutral SNPs after filtering used in the principal components analyses

Between the Central Valley and San Pablo Bay populations, Weir and Cockerham’s pairwise *F*_ST_ was highest for microsatellite data, and slightly higher for neutral SNPs than overall SNPs (Table 2). Gene diversity, heterozygosity, and population-specific *F*_ST_ were all consistent in relationship between the Central Valley and San Pablo Bay populations when compared between the three data sets, with higher values for the Central Valley fish (Table 1). However, *F*_*IS*_ was positive for overall SNPs but negative for neutral SNPs. Moreover, *F*_*IS*_ was indistinguishable from zero for the San Pablo Bay fish when using microsatellites, but was positive for the Central Valley fish using the same data (Table 1). Principal components analysis (PCA) was consistent in separating populations along principal component one (Fig. 1).

**Table 1:** Population genetic results for microsatellites, neutral SNPs, and overall SNPs in two populations of Sacramento splittail (*Pogonichthys macrolepidotus*)

Dataset	Statistic	Central Valley	San Pablo
Microsatellites (*n* = 528 and 191; 19 markers)	**Pairwise** ***F***_**ST**_	0.04262 (0.0296–0.0589)	
	**H**_**O**_	0.605	0.652
	**H**_**S**_	0.622	0.653
	***F***_**IS**_	0.0266 (0.0054–0.0542)	0.00177 (−0.0171–0.0193)
	**Population-specific** ***F***_**ST**_	0.06475 (0.02951–0.1067)	0.01959 (− 0.009956–0.04752)
Neutral SNPs (*n* = 16 per population; 69,951 SNPs)	**Pairwise** ***F***_**ST**_	0.0263 (0.0257–0.027)	
	**H**_**O**_	0.273	0.279
	**H**_**S**_	0.261	0.264
	***F***_**IS**_	−0.0489 (− 0.0515 - -0.0466)	−0.0541 (− 0.0565–0.0517)
	**Population-specific** ***F***_**ST**_	0.0324 (0.0302–0.0348)	0.0170 (0.01504–0.0190)
Overall SNPs (*n* = 16 per population; 420,626 SNPs)	**Pairwise** ***F***_**ST**_	0.0230 (0.0227–0.0233)	
	**H**_**O**_	0.253	0.264
	**H**_**S**_	0.287	0.290
	***F***_**IS**_	0.120 (0.118–0.121)	0.091 (0.0893–0.0924)
	**Population-specific** ***F***_**ST**_	0.03359 (0.03274–0.03450)	0.02213 (0.02121–0.02290)

The Central Valley population (*n* = 191) represents a larger, less salinity-tolerant group than the San Pablo population (*n* = 191). Neutral and overall single nucleotide polymorphisms (SNPs) were generated with raw RNA sequencing data of 32 fish (*n* = 16 per population). There were a total of 420,626 overall SNPs and 69,951 neutral SNPs after filtering. Pairwise *F*_ST_ represents Weir & Cockerham’s pairwise *F*_ST_, H_O_ represents observed heterozygosity, H_S_ represents gene diversity (sometimes referred to as expected heterozygosity), *F*_IS_ refers to the inbreeding coefficient, and population-specific *F*_ST_ refers to a coalescent approach to *F*_ST_. 95% confidence intervals are provided where possible in parentheses, based on 1000 bootstrapping iterations

### Signatures of selection

Using pcadapt on the overall SNPs with no prior information, 659 SNPs showed signatures of selection along principal component one (*q* < 0.05). Using Bayescan on the same set of SNPs with population of origin provided, 155 SNPs showed signatures of selection between the Central Valley and San Pablo populations (*q* < 0.05). Of these SNPs, 98 showed significant signatures of selection in both pcadapt and Bayescan within 75 different transcripts.

### Transcript quantification

Between-population DGE showed 0 transcripts with significant DGE at hours 0 and 72, and 1757 significant genes at hour 168 (*q* < 0.05). Intrapopulation DGE in the Central Valley fish showed 67 genes with significant DGE between hours 72 and 0, 12 significant genes between hours 168 and 72, and 71 significant genes between hours 168 and 0. Intrapopulation DGE in the San Pablo fish revealed 135 genes with significant DGE between hours 72 and 0, 45 significant genes between hours 168 and 72, and 220 significant genes between hours 168 and 0.

Between-population DEU showed 15 genes with significant DEU at hour 0, 2 genes at hour 72, and 189 genes at hour 168 (*q* < 0.05). Intrapopulation DEU in the Central Valley fish showed 22 significant genes with DEU between hours 72 and 0, 11 significant genes between hours 168 and 72, and 0 significant genes between hours 168 and 0. Intrapopulation DEU in the San Pablo fish showed 22 significant genes with DEU between hours 72 and 0, 2697 significant genes between hours 168 and 72, and 630 significant genes between hours 168 and 0.

No genes were significant in any inter- or intra-population comparison for GEV.

### Combining Phenotypic Plasticity & Signatures of selection

**Fig. 2:**
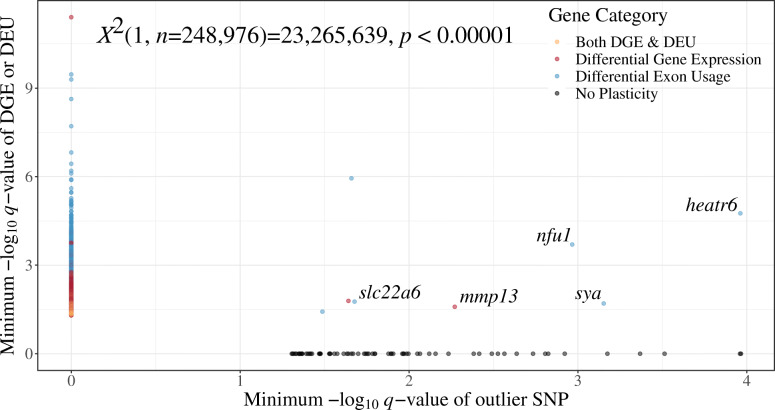
Phenotypic plasticity and signatures of selection in genes plotted by -log_10_ adjusted *p*-values (false discovery rates (*q*)) < 0.05. MRNA data consists of *n* = 16 individuals in each of two populations exposed to a salinity challenge over three timepoints. For visualization, the -log_10_ *q*-value for each gene from differential gene expression (DGE) or differential exon usage (DEU) in any comparison (within or between populations) was drawn and when multiple comparisons were significant (e.g. a gene showing DGE under multiple comparisons), the lowest -log_10_ *q*-value was retained for visualization. Signatures of selection were identified with SNPs that were significant between populations (*q* < 0.05). The lowest significant -log_10_ *q*-value was retained for visualization both between programs and in genes with multiple significant outlier SNPs. 244,089 genes showed no selection or plasticity, 67 showed selection and no plasticity, 4812 showed plasticity and no selection, and 8 showed both plasticity and selection. A *Χ*^2^ test of independence revealed independence between phenotypic plasticity and signatures of selection among genes *Χ*^2^(1, *n* = 248,976) = 22,651,453, *p* < 0.00001. Of the eight genes that show both signatures of selection and phenotypic plasticity, five had available annotations. *HEAT repeat-containing protein 6* (*heatr6*), *NFU 1 iron-sulfur cluster scaffold homolog (mitochondrial)(nfu1)*, *alanine-tRNA ligase (sya)*, and *solute carrier family 22 member 6 (slc22a6)* each show DEU, while *collagenase 3* (*mmp13*) shows DGE

Using the pcadapt and Bayescan outlier results in conjunction with DGE, DEU, and GEV per gene, 67 genes showed only selection, and no plasticity. 4880 showed plasticity and no signatures of selection. Eight had both signatures of selection and plasticity, and 244,021 showed neither selection or plasticity. A *Χ*^2^ test revealed that selection or plasticity are likely to be expressed in different genes *Χ*^2^(1, *n* = 248,976) = 23,265,639, *p* < 0.00001 (Fig. 2).

Patterns of phenotypic plasticity and selection, when plotted with -log_10_ *q*-values, showed an independence between the categorical variables consistent with the *Χ*^2^ test (Fig. 2). Many genes show either signatures selection or plasticity, but not both, whereas eight transcripts show both signatures of selection and phenotypic plasticity. Among the eight genes showing both selection and plasticity, DEU contributed to plasticity in six, whereas DGE contributed plasticity in two. Within phenotypic plasticity, six of eight genes presented significant DEU between the 168- and 72-h timepoints in the San Pablo fish, with no other plasticity expressed by those genes. One remaining gene showed DGE between the Central Valley and San Pablo populations at 168 h (− 0.64 log_2_-fold change, *q* = 0.016), and the other showed DGE within the San Pablo population between 72 and 0 h (− 3.92 log_2_-fold change, *q* = 0.026).

**Fig. 3:**
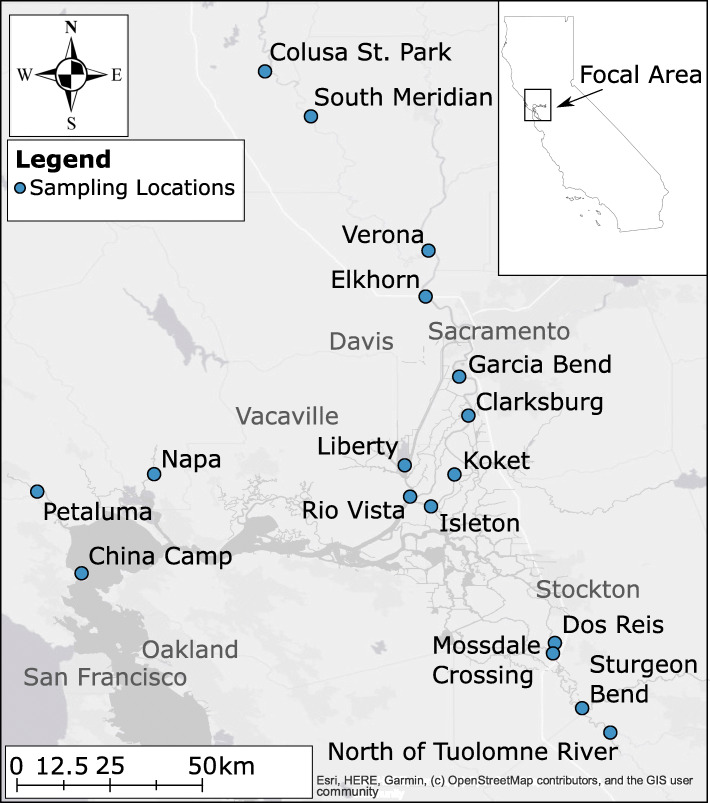
Map of sampling locations used for individuals analyzed in microsatellite-based analyses for Sacramento splittail (*Pogonichthys macrolepidotus*) from the San Francisco Estuary, California, USA. The three western sites comprised the San Pablo Bay region of sampling, while sites east of Fairfield comprised the Central Valley region of sampling. Population reassignment with discriminant analysis of principal components with two clusters placed one cluster of which most individuals were caught in the San Pablo Bay region, and another cluster spread out among both regions of sampling (see Table 2 for details). As such, for microsatellite-based analyses, the individuals placed in the region characterized by the San Pablo Bay region are referred to as the San Pablo Bay population, and other individuals are referred to as the Central Valley population

Of the eight genes that showed both phenotypic plasticity and signatures of selection, five had available annotations. *HEAT repeat-containing protein 6* (*heatr6*), *NFU 1 iron-sulfur cluster scaffold homolog (mitochondrial)(nfu1)*, *alanine-tRNA ligase (sya)*, and *solute carrier family 22 member 6 (slc22a6)* all showed DEU between the 168- and 72-h timepoints within the San Pablo population of fish (Fig. 3). *Collagenase 3* (*mmp13*) also showed plasticity within the San Pablo population, with − 3.92 log_2_-fold change (*q* = 0.026) between the 72- and 0-h timepoints.

Analyses of DGE and outlier SNPs separately are provided in [8]. Functional analyses for DEU are provided in detail in the Supplementary Materials. Briefly, two significant genes with annotations were available for the between-population comparison at hour-0, none were available at hour-72, and 75 genes with annotations were available at hour-168 (Supplementary Table S1). Within Central Valley fish between hours 72 and 0, six significant genes had annotations available, none had annotations available between hours 168 and 72, while one had annotations available between hours 168 and 72. Within San Pablo fish, nine significant genes had available annotations between hours 72 and 0, 298 had available annotations between hours 168 and 0 (Supplementary Table S2). Between hours 168 and 72 in San Pablo fish, 1319 significant genes had annotations available (Supplementary Table S3), with two significant GO terms found using the GO Biological Process 2018 database with EnrichR: golgi vesicle transport (GO:0048193, *q* = 0.014) and protein modification by small protein conjugation (GO:0032446, *q* = 0.015).

## Discussion

Our data show that SNPs called from mRNA are consistent with microsatellite data for describing population differentiation, although the magnitude of differentiation (i.e., *F*_ST_) is lower with mRNA. Moreover, an analysis of genes that show phenotypic plasticity and contain signatures of selection revealed that a given gene is likely to show either selection or plasticity—but rarely both. Patterns of phenotypic plasticity revealed by DEU but not DGE, especially between the 168- and 72-h timepoints in the relatively more salinity-adapted San Pablo fish, confirmed that mRNA is useful for different types of expression quantification-based analyses. Overall, the consistency in signals of population differentiation and the breadth in analyses of phenotypic plasticity possible with mRNA sequencing support its usefulness within the context of both population genetics and phenotypic plasticity.

### Population genetics

Measures of population differentiation and genetic variation were largely consistent between mRNA SNPs and microsatellites, with one important difference. When both filtered for putatively neutral markers and with the overall SNP data, mRNA sequencing revealed pairwise *F*_ST_ approximately 40% lower than *F*_ST_ described using microsatellites. This lower *F*_ST_ described when using mRNA is consistent with lower gene diversity (i.e., expected heterozygosity, H_S_) and heterozygosity relative to microsatellites. Lower gene diversity and heterozygosity may be a result of widespread purifying selection throughout the Sacramento splittail’s transcriptome, a phenomenon hypothesized to exist in mRNA across taxa because of its functional role in organism’s life histories—as opposed to neutral microsatellites [7]. Selection may operate even on synonymous mutations in mRNA and it may be unlikely that any SNP in mRNA is ‘truly neutral’ [5–7]. In addition, reduced heterozygosity and gene diversity may be influenced by lower sample sizes in mRNA data, where sequencing costs may preclude sample sizes often used in microsatellite-based studies. Nevertheless, pairwise *F*_ST_ found using mRNA described two populations consistent with population structure found in other research [32]. In addition, heterozygosity and gene diversity within populations were consistent between mRNA and microsatellites in their relative magnitudes, with slightly higher values in the salinity-tolerant San Pablo fish in each case. Population-specific *F*_ST_ values were also consistent between methods, with lower values in San Pablo Sacramento splittail relative to individuals from the Central Valley using both mRNA and microsatellite markers. Lower *F*_ST_ in this circumstance is related to coancestry and may imply the San Pablo fish more closely resemble the population of origin for the Sacramento splittail [35, 36].

### Phenotypic Plasticity & Signatures of selection

Analyses of signatures of selection and phenotypic plasticity expressed by genes within the context of local adaptation and adaptive responses may elucidate some of the mechanisms by which organisms respond to changing environments. Different perspectives exist on the role of genetic variation on plastic responses. From one perspective, plastic traits may be studied as a morphological or phenological trait such as flowering time or growth rate [17]. From another perspective, plasticity can be represented by environmentally responsive loci [20, 21], a perspective adopted in the present study. Here, the divergent evolution of plasticity plays a role in adapting to environmental change (climate change in [21]; salinity differences in the present study). Prior work showed results consistent with the role of divergent plasticity in the Sacramento splittail, with greater transcriptome plasticity and salinity tolerance observed in the San Pablo population [8, 34]. Consistent with our hypothesis that phenotypic plasticity would diverge from adaptive variation within genes, positive selection or phenotypic plasticity were found in almost mutual exclusion. That is, a gene with signatures of selection between the two populations was unlikely to show any kind of phenotypic plasticity, and a gene showing any intra- or inter-population plasticity in expression was unlikely to have signatures of selection.

The near mutual exclusion of plasticity and signatures of selection shown in the present study is in line with work showing an inhibitory relationship between the two phenomena [20, 21, 37]. Nevertheless, several studies have described a co-occurrence of plasticity and selection at environmentally-responsive genes, such as salinity tolerance genes that may be the targets of adaptive variation in Atlantic killifish (*Fundulus heteroclitus*) [38–40]. The discordance between these results on the relationship between selection and plasticity may have arisen from the evolutionary backgrounds of the plastic traits under study. Killifish have adapted to wide salinity gradients with extreme physiological plasticity [39], whereas the Sacramento splittail is experiencing more variable salinity in the modern day due to many anthropogenic and climate change-related impacts in the system and may have evolved in a more stable saline environment. Therefore, selection may act upon plastic genes in populations extremely tolerant to a stressor, but plasticity may constrain evolution in populations of moderate tolerance to a stressor. These findings are consistent with the Sacramento splittail having evolved at a fitness peak where high levels of plasticity in the San Pablo Bay population reduce the likelihood of genetic change with respect to salinity tolerance because plasticity itself has undergone selection [41]. Any mutations in the genes that compromise the plastic response are likely to be deleterious if the San Pablo population is at a fitness peak, and purifying selection may be a major force in these plastic pathways.

Among the five genes that contained signatures of selection and phenotypic plasticity and were also annotated, four showed DEU between the 168- and 72-h timepoints in the San Pablo population of Sacramento splittail exposed to salinity (*heatr6*, *nfu1*, *slc22a6*, *sya*). These genes may therefore exhibit differential splicing in response to salinity and in conjunction with the signatures of selection within them, may be important components of local adaptation in the San Pablo population. The San Pablo fish have shown a more plastic, likely adaptive response to salinity challenge than the Central Valley fish overall [8, 33, 34]—a response recapitulated in the novel patterns of DEU described in the present study. Alternative splicing, that leads to the DEU, has been discussed in fish in evolutionary and physiological contexts, with roles in heat stress, cold acclimation, jaw morphology, and mate choice, with implications for adaptive radiations [24–27]. It is therefore unsurprising that DEU plays a role in the Sacramento splittail’s response to salinity because of the salinity differences in the fish’s native environment [32]. However, the novel patterns of DEU in response to salinity in the Sacramento splittail, in conjunction with the genes that showed both DEU and selection, is consistent with adaptive roles for DEU in both physiological environmental responses and functional evolutionary differences among populations [42].

## Conclusions

We described applications of mRNA sequencing for delineating population structure and investigating dynamics between plasticity and selection. Our measures of genetic variation and population differentiation were consistent with previously hypothesized purifying selection across organism’s transcriptomes [7]. In practice, this purifying selection may have led to lower gene diversity, heterozygosity, and *F*_ST_ estimates found with mRNA relative to microsatellites in these data. Population genetic measures drawn from mRNA data must therefore be interpreted with caution (and as conservative estimates) when used for characterizations of population structure, especially for studies with management implications. MRNA sequencing also provides fertile ground for studying the relationship between phenotypic plasticity and selection, within a mechanistic framework. While a wide body of research on the question describes phenotypic plasticity in non-molecular terms (e.g. bloom timing, salinity tolerance), mRNA data describes phenotypes by the expression of individual transcripts or genes. By quantifying the expression of individual transcripts, aligning transcripts to gene representations, and investigating outlier SNPs, researchers can use mRNA data to find key information about molecular mechanisms underlying local adaptation and adaptive responses to changing environments.

## Methods

### Data sets

The microsatellite data set used for the present study was published in [33]. Briefly, *n* = 727 individuals collected in 2011 and 2012 from six sites representing the San Pablo Bay and Central Valley splittail populations were analyzed [32, 33]. The San Pablo Bay population was represented by individuals collected from the Napa River, Petaluma River, and in San Pablo Bay itself (*n* = 119, 293, and 3, respectively) (Fig. 3). The Central Valley population was represented by individuals collected from Liberty Island, the Sacramento River, and the San Joaquin River (*n* = 49, 128, and 135, respectively) (Fig. 3). Nineteen microsatellites previously described were used, and individuals with 20% or greater missing data were removed (≥3 microsatellite loci missing) [43]. Population reassignment was performed using Adegenet version 2.1.2 with 75 principal components and two clusters [44, 45]. To address the possibility that family structure may bias measurements of population structure, Colony version 2.0.6.5 was run separately on each of the reassigned clusters [46]. In Colony, allele frequencies were updated, inbreeding was allowed, polygamy was allowed for males and females, full sibship scaling was used, a weak sibship prior was assumed, and full-likelihood-pair-likelihood combined scores were used at high precision over 10 replicate runs in each cluster. Individuals were considered full-siblings for removal with an inclusive probability > 0.80 for the pairing. Cluster 1 consisted of *n* = 531 individuals from all six sites with 3 individuals removed as full-siblings of others, while Cluster 2 consisted of *n* = 196 individuals with 5 individuals removed as full-siblings of others, primarily from the San Pablo Bay (Table 2). Hereafter, Cluster 1 will be referred to as the Central Valley population while Cluster 2 will be referred to as the San Pablo Bay population with respect to the microsatellite data.

**Table 2:** Sacramento splittail (*Pogonichthys macrolepidotus*) sample sizes for individuals used in microsatellite data by region, capture location, and population reassignment

Region	Location	Cluster One	Cluster Two
San Pablo Bay	Napa River	62	56
	Petaluma River	169	117
	San Pablo Bay	3	0
Central Valley	Liberty Island	47	2
	Sacramento River	123	5
	San Joaquin River	124	11
Total		528	191

Region describes the overall region of capture, within which are rivers and capture sites at which fish were collected described by Location. Clusters One and Two describe population reassignment, where Cluster One is comprised of individuals across all six capture locations, while Cluster Two is comprised of individuals from capture locations primarily in San Pablo Bay. Throughout the present manuscript and with respect to microsatellite data, Cluster One is referred to as the Central Valley population and Cluster Two referred to as the San Pablo Bay population. In Adegenet, 75 principal components and two clusters were chosen for analysis

The mRNA data set used for the present study was published in [8], where *n* = 16 fish from each the San Pablo Bay and Central Valley populations of Sacramento splittail were exposed to a salinity challenge of 14 PSU. Fish were sacrificed and gill tissue was sampled 0, 72, and 168 h into the salinity exposure (see [8] for details). In the present study, the raw reads were downloaded from the National Center for Biotechnology Information Sequence Read Archive (accession #PRJNA326543) and the SuperTranscripts pipeline was used to align raw reads to a published reference transcriptome because of its capacity for describing DEU in non-model organisms [8, 30]. Following the SuperTranscripts pipeline, Salmon version 0.11.3 was used for quasi-mapping prior to clustering transcripts using Corset version 1.07 [47, 48]. These Corset-clustered reads were used for expression quantification-based approaches used in this study (i.e. differential gene expression, differential exon usage, and gene expression variation). From the Corset-clustered reads, a linear representation of the transcriptome was created using Lace version 1.00 [30]. Final alignments were performed with STAR version 2.7.0a [49]. Throughout the present manuscript, the Corset-clustered SuperTranscripts are referred to as “genes.”

SNPs were called from STAR-aligned reads and the Lace-reconstructed transcriptome by adding read groups, splitting cigar ends, and merging bam files with Picard version 2.18.9, then using FreeBayes 1.2.0 for final SNP calling [50, 51]. Here, 3,284,734 SNPs and indels were called with FreeBayes. SNP filtering was done using VCFtools version 0.1.14 [52]. From the initial data set, 420,626 high-quality SNPs was created by filtering to include only biallelic SNPs of genotype and site qualities > 30, minor allele frequencies of ≥0.05, and a maximum of 20% missing data. Because the markers used in the microsatellite data set described above were in HWE, another set of SNP data was created using vcftools, with genotype and site qualities of 30, minor allele frequency of ≥0.05, biallelic SNPs, no missing data, and within HWE at *p* < 0.005. These SNPs were then pruned for linkage disequilibrium (LD) using SNPRelate version 1.16.0 at a threshold of 0.20 [53]. SuperTranscript clusters were coded as chromosomes for the purposes of LD pruning [53]. After pruning for LD, 69,951 SNPs remained. Hereafter, the SNP data set filtered for quality but *not* HWE or LD is referred to as “overall SNPs”, while the SNP data set filtered for HWE and LD is referred to as “neutral SNPs.”

### Population Structure & Genetic Variation

To examine how well SNPs from mRNA recapitulate patterns of genetic variation and population structure revealed by microsatellites, Hierfstat version 0.04–22 [4] was used to evaluate pairwise Weir and Cockerham’s *F*_ST_, along with population-specific *F*_ST_, observed gene diversity, and *F*_IS_. These tests were performed on each of the three data sets: microsatellites, overall SNPs, and neutral SNPs. For statistics calculated in Hierfstat, 95% confidence intervals calculated using bootstrapping over 1000 iterations. Population structure was visualized using principal components analysis (PCA) as implemented in Adegenet version 2.1.2 [44].

### Signatures of selection

Two different programs were used to analyze signatures of selection, pcadapt and Bayescan [54, 55]. In each of these programs, the overall SNP data set of 420,626 SNPs was used. For pcadapt version 4.3.3, two principal components were used, and samples separated by population along principal component 1 (PC1), which explained 25.4% of the variance in the data. *P*-values for all SNPs were adjusted with a false discovery rate (*q*) correction for multiple tests, then SNPs with a *q* < 0.05 that varied along PC1 were kept.

### Transcript quantification

Three transcript quantification-based methods were used to analyze mRNA expression data from [8]: differential gene expression (DGE), differential exon usage (DEU), and gene expression variability (GEV). From the transcript reads clustered with Corset, DGE was analyzed using edgeR version 3.28.1 [56]. Data were filtered for any transcript expression within any of six groups (i.e., a transcript was retained only if all individuals in at least one group showed expression at that transcript); out of 248,976 transcripts, 68,737 were kept in this way. After estimating dispersion, generalized linear models with quasi-likelihood tests were used to estimate DGE between populations at each of three experimental timepoints, and within populations between the three experimental timepoints. Only genes with *q* < 0.05 for DGE were kept for downstream analyses.

Exon counts for DEU were estimated with the featureCounts function of Lace, version 1.00. These exon counts were then analyzed for DEU with edgeR version 3.28.1 and Limma version 3.42.2 [29, 56]. Briefly, normalization factors were calculated, observation-level weights were computed with voom, linear models were fit for each exon, then DEU was tested with diffSplice. Pairwise comparisons were drawn between two populations at each of three experimental timepoints, and within populations between each of three experimental timepoints. Only genes showing DEU with *q* < 0.05 were kept for downstream analyses.

Code provided in [31] was modified to calculate GEV between two populations at three experimental timepoints, and within populations at each experimental timepoint. Normalization factors were calculated with edgeR version 3.28.1, then offset variables were calculated as the natural log of the product of library size and normalization factor [56]. Only genes with greater than one count per million were included in the analysis. The R package GAMLSS version 5.1–6 was used to estimate GEV with the resulting data sets [57]. First, a negative binomial model that included groups of interest and offset variables was fit. Then, group factors were omitted from estimations of mean and overdispersion in expression, respectively, along with a null model fit with just the offset variables. Estimations of non-Poisson noise were tested with a log-likelihood ratio test in GAMLSS, then Corset-clustered reads with inflated or near-Poisson coefficients of variation (CV) in mRNA copy number were removed (1 × 10^− 3^ < CV < 3). Last, false discovery rate adjustments were calculated with reported *p*-values for CV. Only genes with *q* < 0.05 were kept for downstream analyses.

### Combining Phenotypic Plasticity & Signatures of selection

A chi-square test of independence was used to explore the relationship between signatures of selection and phenotypic plasticity shown by individual genes. Here, a transcript was counted as showing signatures of selection if it contained a significant outlier SNP between populations (*q* < 0.05) as identified by pcadapt and Bayescan, or counted as exhibiting phenotypic plasticity if significant DGE, DEU, or GEV (*q* < 0.05) was identified in the transcript. Transcripts showing neither selection or plasticity were also counted.

Different types of phenotypic plasticity (all comparisons within DGE, DEU, and GEV) were summarized at the gene level by first identifying the types of significant (*q* < 0.05) plasticity within a gene, then identifying the lowest -log_10_ *q*-value among the different types of plasticity, if more than one was present for a transcript. If only one type of plasticity was present in a gene, the associated log-transformed *q*-value was associated with overall plasticity for the gene. Similarly, −log_10_ *q*-values were calculated for each significant outlier SNP found using Bayescan or that varied along PC1 using pcadapt (*q* < 0.05). Within a gene, the minimum significant log-transformed *q*-value was identified, and that value was associated with signatures of selection for the entire transcript for plotting. Genes were thus represented by four categories: those showing no signatures of selection or plasticity, those showing only selection and no plasticity, those showing only plasticity and no selection, and those showing both plasticity and selection.

Functional analyses of genes under different conditions of selection, plasticity, or both were analyzed using the annotated transcriptome used in [8]. Because patterns of DGE and selection were analyzed in prior research, analysis of DEU, GEV, and of genes showing overlapping plasticity and selection are focused on, here [8]. A detailed description of gene set enrichment analysis in genes showing DEU using EnrichR [58] is provided in the Supplementary Materials.

## Supplementary Information

**Additional file 1: Supplementary Table S1**. Differential exon usage (DEU) at 168-h between the Central Valley and San Pablo populations of Sacramento splittail (*Pogonichthys macrolepidotus*). Only the 75 of 189 genes that showed significant DEU in this comparison and had annotations are included here, sorted by ascending false discovery rate-adjusted *p*-values (*q* value). **Supplementary Table S2**. Differential exon usage (DEU) between hours 168 and 0 for the San Pablo San Pablo population of Sacramento splittail (*Pogonichthys macrolepidotus*). 630 genes showed significant DEU in this comparison, but only the 199 with annotations are included here, sorted by ascending false discovery rate-adjusted *p*-values (*q* value). **Supplementary Table S3**. Differential exon usage (DEU) between hours 168 and 72 for the San Pablo San Pablo population of Sacramento splittail (*Pogonichthys macrolepidotus*). 1319 genes with annotations are included here, sorted by ascending false discovery rate-adjusted *p*-values (*q* value).

## Data Availability

All code used in the present study is provided at https://github.com/BioMatt/splittail_msat_RNA. The raw mRNA reads from [8] supporting the conclusions of this article are available through the National Center for Biotechnology Information Sequence Read Archive (accession #PRJNA326543; https://www.ncbi.nlm.nih.gov/bioproject/PRJNA326543). The microsatellite dataset from [33] is available on GitHub (https://github.com/BioMatt/splittail_msat_RNA/blob/master/microsatellites/splittail_msat_data.txt).
